# Notes and News

**Published:** 1891-10-17

**Authors:** 


### Notes and News.

COLLECTED AND COMMUNICATED.

At a general court of Governors of the Seamen's Hospital Society it was reported that the society had opened a dispensary at 51, East India Dock Road. The general work of the society has been carried on with much vigour during the last quarter. The number of patients at the Dreadnought Hospital, Greenwich, and at the branch hospital, Royal Victoria and Albert Docks, has been greater than in any former period. The patients treated represent 27 nationalities, including English, Swedes, Norwegians, French, Germans, Austrians, &c. With the extended work further funds are urgently needed on behalf of this national charity. Sir Frederick Young, K.C.M.G., has been elected a member of the Committee of Management.

The National Health Society has again commenced its winter campaign by organizing special courses of lectures on sanitation, domestic and personal hygiene, elementary anatomy and physiology, nursing, accidents and disease. These lectures will be given in the lecture room of the society, 53, Berners Street, W., by the society's competent staff of lecturers, commencing on October 13th at five p.m. (an hour suitable to those engaged during the day), and they will be of a specially practical nature, and most suitable for those leaving school being a most important and necessary course of study for all. Each lecture will be fully illustrated by interesting diagrams and models. For syllabus and other particulars application may be made to the Secretary, 53, Berners Street, W.

A correspondent of the *Medical Press and Circular* writes that the Austrian Minister of the Interior has promulgated a law requiring that all medical prescriptions be clearly and legibly written, so that there can be no doubt as to the medicine ordered, the amount to be taken, the directions for the patient, and the prescriber's name. Nothing is more essential than legible writing in prescriptions, and in no documents known, is there less care taken to avoid error than in these. If the illegible writing had not occasioned death there might be some excuse for the carelessness in this particular of prescribers. But knowing that a wrong interpretation of the hastily-written and often illegible prescription may have a fatal result, and certainly will interfere with the patient's recovery, not a word by way of justification can be pleaded. The journal does not desire legislative interference in this matter, as in Austria, but it urges on the licensing bodies the advisability of requiring of candidates a good intelligible handwriting, and thinks compounding houses might make it a trade rule to refuse any prescription that was not plainly written.

In a previous number of this paper we drew attention to the mistakes that occurred, and were likely to occur, in dispensing of drugs by apothecaries. It appears that the maladministration of strychnine in place of Epsom salts has occasioned the death of Jennie D'Achu. The jury returned a verdict to the effect that there was not sufficient evidence to show how the poison came into the Epsom salts.

Sister Queen, late of the London Hospital, makes an appeal in aid of the Invalid Children's Association, 18, Buckingham Street, Strand. This Association supplies a much felt want. The change after the careful treatment and good nourishment received at the hospital to the neglect and want of a miserable home, is felt severely by many poor children. To the assistance of such the Association steps in, and, as far as is in its power, provides the most needful aid to the little patients on leaving the hospital. Over 15,000 have already made applications for aid; much good work could be done that is at present impossible, owing to want of funds.

The Congress of Analytical Chemists, which is now assembled here to pave the way to an international agreement for the suppression of the adulteration of articles of food is passing several resolutions of interest. One of these recommends that infants' milk should be subjected to the sterilising process at the dairies. Another declares that the babies' food called, *Kindermehl*, ought not to be given to children before they are six months old. The Congress also occupies itself with articles of the toilet and of perfumery, and a committee was appointed to consider whether sanitary control ought not to be extended to hairdressers' shops and public baths, and whether the daily disinfection of the brushes and combs used in shops and public places ought not to be made obligatory by legislation. Another question under discussion was the desirability of the sanitary control of all publicly advertised soaps, rouge, and other cosmetics.

Professor Macnamara, in the course of his address at the opening of the Winter Session of the Meath Hospital, dwelt especially on the "traps" in the path of the medical student, and said: "The first of these traps is found in the fact that in many instances the choice of a profession is decided for him, irrespective of the young man's vocation. There are those to whom each succeeding day's duty makes their task more repulsive, and to such a one would I give the advice: seek for the talents with which Providence has blessed you in some other outlet more congenial to your tastes. The late Bishop of Peterborough was a medical student for six months in the wards of the Meath Hospital. He abandoned medicine in favour of divinity. The second trap that awaits the student has, I regret to state, been laid for him by the General Medical Council. They have made Greek an optional instead of a compulsory subject, to be passed previous to registration as a medical student. Greek is a more living language than any modern one to a medical practitioner, so many are the terms supplied by it to our nomenclature. Another trap lying in our path is found in our disregard of the advice, "Be not the first by whom the new is tried, nor the last the old to lay aside." New medicines are being forced upon our notice, and but too frequently supplanting older and more valuable medicines of the same class. Surgeons' minds have been exercised in devising operations for the relief of diseases previously considered outside the pale of their art. Physicians have been earnest in their efforts at the elucidation of hitherto obscure, in some instances unrecognised, symptoms. I wish to urge upon your consideration the propriety of exercising a due amount of reflection and caution in accepting as gold that which only glitters. The Professor then goes on to say that lastly the trap set by anti-vaccinators—namely, that disease is generated by vaccination—is to be avoided; he denies their contention that the virus of disease is introduced into the baby's system through or along with the vaccine matter employed, and says further: "When I was a boy, the number of middle-aged people one met disfigured with small-pox was simply appalling; now I challenge my youthful hearers to go beyond the fingers of one hand to enumerate such of their acquaintances as are so disfigured."

